# Supplementary material for: Identification of photoperiod-induced specific miRNAs in the adrenal glands of Sunite sheep (Ovis aries)
Source: Front Vet Sci. 2022 Jul 22;9:888207. doi: 10.3389/fvets.2022.888207 (PMC9354845; doi:10.3389/fvets.2022.888207)
Supplement: Supplementary file 1 [file Data_Sheet_1.ZIP › Supplementary file/miRDeep2 file.zip!/miRDeep2 file/Novel_Structure/LP42A3/Novel_173.pdf]

Provisional ID	:	NW_014639620.1_25590
Score total	:	1.7
Score for star read(s)	:	-1.3
Score for read counts	:	0
Score for mfe	:	1.4
Score for randfold	:	1.6
Score for cons. seed	:	
Total read count	:	47
Mature read count	:	47
Loop read count	:	0
Star read count	:	0

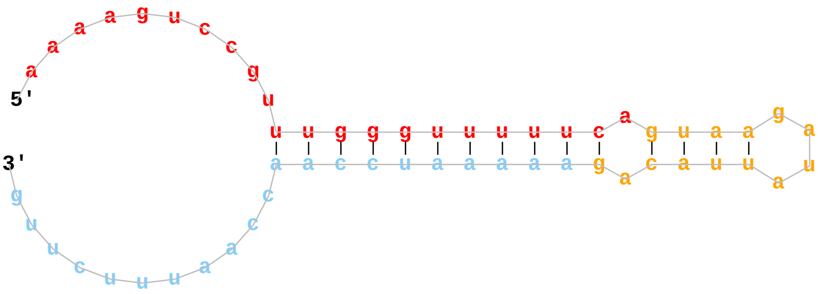

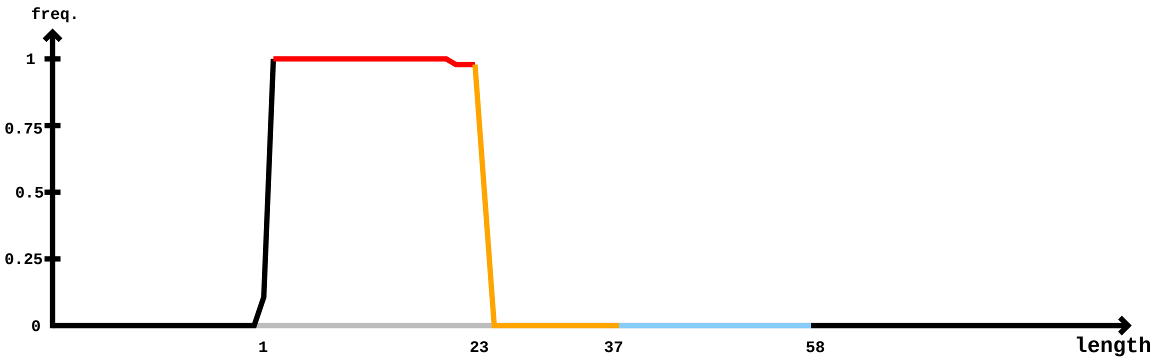

Mature Star

```
5'- auaauauauuggguuggcaaaaaaaguccguuuggguuuuucaguaagauauuacagaaaaauccaaccaauuucuuggccaacccaguacuaugauauauuuaauagcu   -3'    exp
    ......((((((((((((..((.((((..(((.(((((((((.((((....)))).))))))))))))..)))).)).))))))))))))((((..........))))..    reads    mm    sample
    ....................aaaaaguccguuuggguuuu......................................................................    1        0     seq
    ....................aaaaaguUcguuuggguuuuuca...................................................................    4        1     seq
    .....................aaaaguccguuuggguuuuuca...................................................................    1        0     seq
    .....................aaaaguUcguuuggguuuuuca...................................................................    41       1     seq
```
